# Scoliosis treatment using a combination of manipulative and rehabilitative therapy: a retrospective case series

**DOI:** 10.1186/1471-2474-5-32

**Published:** 2004-09-14

**Authors:** Mark W Morningstar, Dennis Woggon, Gary Lawrence

**Affiliations:** 1 Director of Research, Pettibon Biomechanics Institute 3416-A 57^th ^St Ct. NW; Gig Harbor, WA 98335, USA; 2 Director, CLEAR Institute; 437 North 33^rd ^Ave; St. Cloud, MN 56303, USA; 3 Director, Lawrence Chiropractic Clinic, 13961 60^th ^St North; Stillwater MN, 55082, USA

## Abstract

**Background:**

The combination of spinal manipulation and various physiotherapeutic procedures used to correct the curvatures associated with scoliosis have been largely unsuccessful. Typically, the goals of these procedures are often to relax, strengthen, or stretch musculotendinous and/or ligamentous structures. In this study, we investigate the possible benefits of combining spinal manipulation, positional traction, and neuromuscular reeducation in the treatment of idiopathic scoliosis.

**Methods:**

A total of 22 patient files were selected to participate in the protocol. Of these, 19 met the study criterion required for analysis of treatment benefits. Anteroposterior radiographs were taken of each subject prior to treatment intervention and 4–6 weeks following the intervention. A Cobb angle was drawn and analyzed on each radiograph, so pre and post comparisons could be made.

**Results:**

After 4–6 weeks of treatment, the treatment group averaged a 17° reduction in their Cobb angle measurements. None of the patients' Cobb angles increased. A total of 3 subjects were dismissed from the study for noncompliance relating to home care instructions, leaving 19 subjects to be evaluated post-intervention.

**Conclusions:**

The combined use of spinal manipulation and postural therapy appeared to significantly reduce the severity of the Cobb angle in all 19 subjects. These results warrant further testing of this protocol.

## Background

In the MEDLINE- indexed literature, chiropractic treatment has shown to be largely ineffective at significantly reducing scoliotic curvatures. Chiropractic treatment for scoliosis typically consists of spinal manipulation, electric stimulation, some form of isotonic, active exercises, and shoe lifts [1]. However, Lantz et al [2] has shown that these procedures, when applied over a one-year duration, were not sufficient to significantly reduce the Cobb angle of a scoliotic curvature.

The treatment in this study focuses on the reduction of scoliosis by manipulative and rehabilitative methods not commonly used by most chiropractors. The major difference in this treatment compared to others is that stimulation of the involuntary postural reflexes is utilized in the clinic setting as well as in home care. Many of the proposed etiologies of idiopathic scoliosis are neurological in origin, including brain asymmetry [3], neural axis deformities [4], and central nervous system processing errors [5]. Additionally, many coexistent neurological alterations are present in scoliosis patients, such as visual deficiency [6] and decreased postural stability [7,8]. Therefore, the goals of the proposed treatment are not only to reduce the scoliotic curvatures, but also to rehabilitate any underlying postural and neurological weaknesses or imbalances. Previous chiropractic authors have investigated the effectiveness of various physiotherapeutic modalities in the treatment of scoliosis, such as Pilates [9], stretching and massage [10], therapeutic exercises [11], orthotics [2], and ultrasound or electric stimulation [1]. The purpose of the present study is to investigate any possible benefits from combining manipulative and rehabilitative techniques from a randomized sample collected from various chiropractic facilities. Preliminary evidence [12] suggests that these procedures may be beneficial for reducing the curvatures associated with scoliosis.

## Methods

A nonrandomized set of 22 patients participated in the study. The age range of the subject group was 15–65 years of age. The patients were selected from 3 different chiropractic facilities in the United States. Patients were evaluated according to their chief complaint at initial presentation. Patients were excluded from the study if neoplasm, malignancy, fracture, scoliosis secondary to genetic disorders, or previous arthrodesis were identified.

Each patient was examined radiographically for location and severity of scoliosis with standing anteroposterior full spine imaging. All patients removed their shoes for the imaging. Cobb angles were drawn on each radiograph to identify the degree of curvature present. A specific treatment plan was created based upon the results of each patient's radiographic measurements before and after a sample trial of the proposed clinical procedures. Initially, standing lateral cervical, nasium, lateral lumbar, and anteroposterior lumbopelvic views were taken. These views were taken to quantify forward head posture, cervical lordosis, lumbar lordosis, the sacral base angle, and the Cobb angle of the major lateral curvature. We decided to use the radiographic positioning and analysis outlined by Harrison et al [13-16], due to its previously published reliability. After these images were taken, each patient was fitted with a 4-lb anterior headweight. They were instructed to walk around with the headweight for 10 minutes. After 10 minutes, a follow-up lateral cervical radiograph was taken while wearing the anterior headweight. The purpose of this lateral stress view is to evaluate the potential improvement in cervical lordosis and reduction in forward head posture from using these procedures [17,18]. The basis for this aspect of the protocol is based upon the inherent properties of a curved column. In the spine, lateral spinal displacements may occur when the normal sagittal spinal curves [19-22] are flattened, reversed, or accentuated. These curves are necessary for the overall strength and flexibility of the curved spinal column, according to the Delmas Index [23]. Therefore, the proposed treatment is intended to restore a normal cervical and lumbar lordosis, and reduce forward head posture before the scoliotic curvatures are addressed.

The specific manipulative and rehabilitative procedures used in this study are designed to both reduce the scoliotic curvature and theoretically retrain the involuntary neuromuscular, reflexive control of posture and balance. However, the specific neurological effects, if any, remain to be investigated. Some of the procedures have been separately introduced or tested [17,18,24-26].

**Figure 1:**
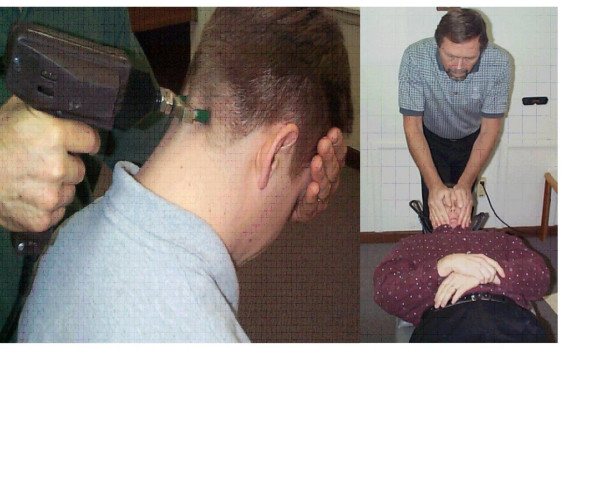
The picture on the left demonstrates the mechanically assisted manipulation used when a patient's skull is restricted in extension on lateral cervical radiograph. The picture on the right is the procedure used when the skull flexion is restricted.

The manipulative procedures included an upper cervical adjustment designed to mobilize the atlantal-occipital joint with the use of a percussive instrument. This technique is shown in Figure 1. This technique is delivered to patients whose lateral cervical radiographs demonstrated atlanto-occipital flexion. If atlanto-occipital extension was present on the initial lateral cervical radiograph, a -Z drop piece was used to mobilize the occiput into flexion. This is also shown in Figure 1. An anterior thoracic adjustment was administered with the patient's thoracic cage rotated opposite to the rotational displacement. A thoracic drop piece was also used to mobilize and correct the smaller upper thoracic curvature. Side posture lumbopelvic adjustments were delivered bilaterally to correct the rotational component of the pelvic misalignment. These side-posture manipulations were performed on a 30°-incline bench to help pre-stress the spine out of its existing scoliotic curvatures.

**Figure 2:**
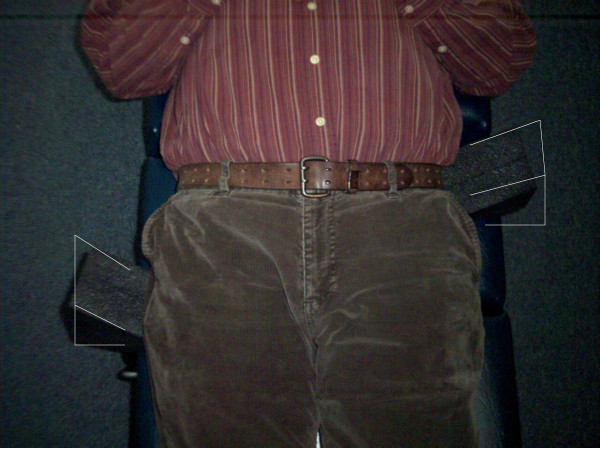
This picture shows the placement of the pelvic blocks for an anterior right ilium. The blocks are placed opposite of the pelvic rotation.

**Figure 3:**
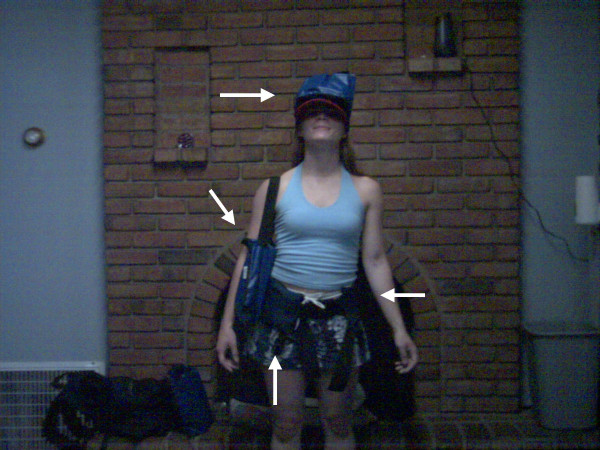
The above picture illustrates a sample placement of the Pettibon Bodyweighting System. Here we have an anterior headweight, right shoulderweight, and left-back and right-front hipweights.

Certain traction procedures are also employed. These procedures are delivered using high-density foam blocks to pre-stress the spine into specific positions so ligament deformation and stress-relaxation can take place. Supine pelvic blocking was performed on each patient for 15 minutes. The position of the blocks was determined by each patient's pelvic rotation on radiograph and posture analysis. One block is placed under the iliac crest of the posterior ilium, and the other block is placed under the femoral head of the opposite, anteriorly-rotated ilium. Figure 2 illustrates the position of the pelvic blocks. The rehabilitative procedures, demonstrated in Figure 3, included the use of head, shoulder, and hip weighting devices. These devices may be used while simultaneously performing specific balancing exercises. These exercises include the use of a Pettibon Wobble Chair^® ^and a Posturomed^® ^[17]. Tjernstrom et al [27] showed that repeated performance of a postural alteration induces a long-term motor memory for achieving that novel postural position.

The position of the body weighting was also determined radiographically for each patient. Initially, hipweights and shoulderweights were applied according to each patient's posture analysis. Anteroposterior cervicothoracic and lumbopelvic views were taken while wearing the head and body weighting. Since changes in spinal position are not reliably seen by visualization [28,29], these stress radiographs were taken to confirm their corrective effects.

The attending physician treated each patient 3 times per week for the first 4–6 weeks. A total of 3 physicians performed the treatment intervention for all patients. However, each patient did not receive identical treatment at all visits. The physicians performed only those manipulative procedures that were deemed necessary based upon a visual posture analysis at the beginning of each treatment session. However, the rehabilitative procedures remained constant throughout the study for all patients.

**Figure 4:**
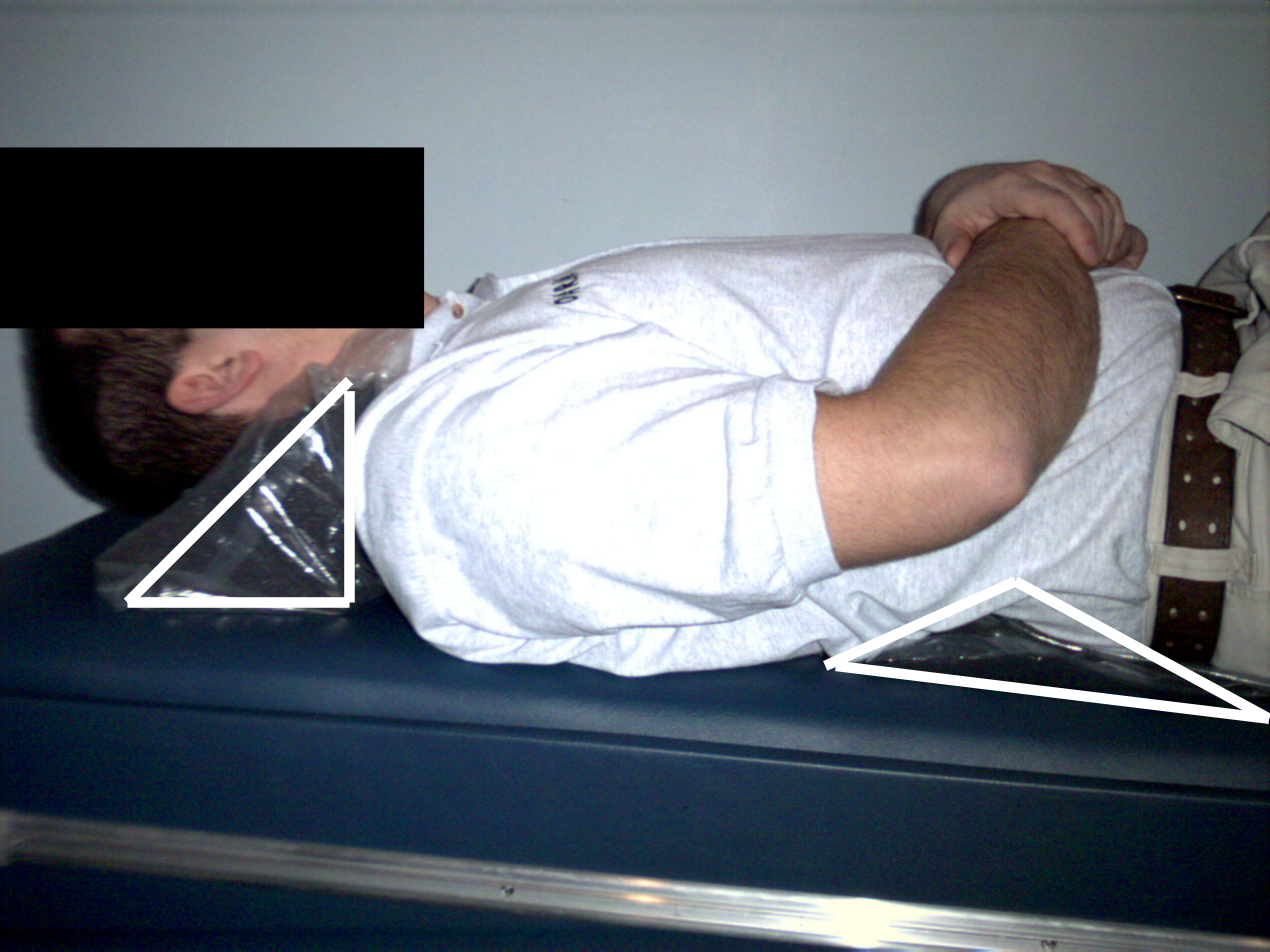
This figure shows a demonstration of the positional traction procedure. The cervical block is placed under the patient's cervicothoracic junction, allowing the head to extend back over the sloped portion of the block. The low back support is placed under the patient's thoracolumbar junction, posterior to the lowest palpable ribs. The blocks are outlined in white.

Specific home care exercise programs were taught to each patient. These exercises were performed on a daily basis. Each patient was instructed to wear the head and body weighting twice daily for 15 minutes each time. Secondly, each patient was given a set of triangular foam blocks to lie on once daily for 20 minutes, immediately prior to going to bed at night. The foam blocks were positioned under the cervicothoracic and thoracolumbar regions simultaneously. The position of these blocks is shown in Figure 4. Patients participating in any weightlifting activities were required to cease those activities until further notice from the attending physician. Patients who failed to perform the home care more than 3 times were dismissed from the study. A total of 3 subjects were eventually dismissed, leaving 19 subjects to perform post-intervention evaluations.

## Results

**Figure 5:**
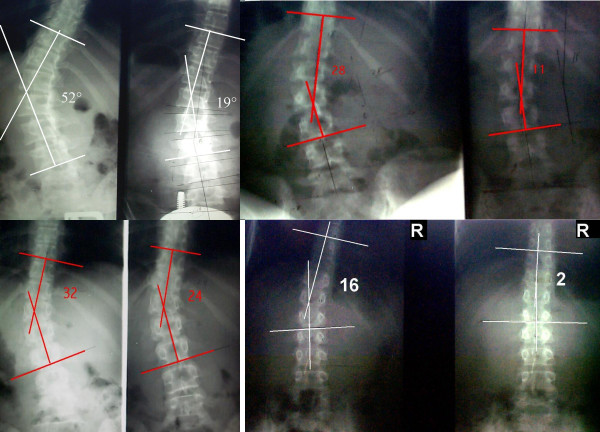
This figure illustrates some of the pre and post x-rays taken after 4–6 weeks of treatment.

At the conclusion of the trial period, a post-intervention radiographic study was conducted. The same anteroposterior full spine view was taken, and Cobb angles were again measured at the same vertebral levels. The average starting Cobb angle was found to be 28°, while the post-intervention Cobb angles measured an average of 11°, for an overall average reduction of 17°. Every patient made at least a 25% improvement. The largest improvement measured 33°, and the smallest improvement measured 8°. Table 1 shows the results of all 19 patients that followed through with the entire treatment plan. Figure 5 is a sample of the improvements made by a few of the patients.

It is important to mention that these patients were initially treated prior to this study. Because of this, the pre and post treatment radiographs had previously been analyzed for sagittal curve and Cobb angle measurements. For purposes of this study, however, all of the radiographs were sent to a single chiropractic physician to analyze each of the patient files. This physician did not participate in the treatment process, nor did this physician have contact with any of the patients. This was performed to separate examiner bias from the treatment results.

While only radiographic procedures were reported for this study, other physiologic parameters were utilized to document patient progress. Unfortunately, since the patient files were extracted from 3 different spine clinics, a consistent functional or symptomatic measure was not used in all 22 cases. A functional rating index, a visual analog scale, and SF-36 were used on the patients here. As a result, these values are not reported to avoid variability in outcome interpretation.

## Discussion

Scoliosis has recently been associated with a lower quality of life [30-32], lower scores on the SF-36 health questionnaire [33], and makes patients prone to developing chronic pain more often than the general population [34]. Therefore, reducing scoliotic curvatures, even in the absence of symptoms, seems to be a worthy outcome objective for clinical practice. This opinion is further supported by recent evidence of the deleterious effects of abnormal spinal loading [35-37]. Given that the average curvature progression in idiopathic scoliosis is 7.03° per year [38], the traditional method of regular observation without treatment seems to be reactionary rather than corrective or preventive.

Spinal manipulation alone does not appear to significantly alter spinal structure when administered as a sole treatment modality [39,40]. Therefore, in the instance of scoliosis, treatment should include the use of both manipulative and rehabilitative procedures, so that structural changes can be attempted. It is important to stress that spinal manipulation was avoided, when possible, in the present study. Unpublished clinical observation by the authors has shown that over-manipulating or adjusting the spine seems to create a certain amount of instability, possibly leading to further buckling of the scoliotic curvature. The significance of home care to the results was not reported here. It is unknown how the omission of home care would have affected the outcome measurements, given that 3 subjects were dropped from the study for noncompliance in performing home care. Future research should account for this potential variable to determine its necessity and relevance.

The outcome measures for this study are divided into a series of both short-term and long-term goals. The outcome of the initial stage of care is to reduce forward head posture and improve the sagittal cervical and lumbar curves. As the position of the head migrates forward, or away from the body's vertical axis, increased strain is placed upon the muscles of the head, neck and shoulders. Cailliet and Zohn indicated that an additional 10 inch/lbs of leverage is added to the spinal system in a forward head posture [41,42]. Additionally, this added leverage causes increased isometric contraction of various spinal muscles, such as the splenius capitis, trapezius, SCM, and levator scapula. Sjogaard et al [43] reported that blood flow through a given muscle is decreased as a muscles contraction increases, being virtually cut off at 50–60% contraction. The resultant lack of blood flow forces the muscle to rely on anaerobic metabolism. As anaerobic metabolism progresses, metabolites such as substance P, bradykinin, and histamine build up and excite chemosensitive pain receptors, causing a barrage of nociceptive afferent input [44], resulting in dysafferentation [45]. Being that postural control is largely dependant upon cervical joint mechanoreceptors and afferent input from ligament and musculotendinous sources [46,47], correcting the postural distortions responsible for this pathophysiologic process may be beneficial in patient populations, such as scoliosis, where postural control is significantly altered [48].

The effects of the loss of cervical and lumbar lordosis have been previously reported [19,35-37]. Rhee et al [49] noted that correction of the sagittal curves might be related to the long-term health of the spine in scoliosis management. Harrison et al [35] illustrated how a loss of the sagittal curve alters the mechanical properties of the spinal cord and nerve roots, which may change the firing patterns of involved neurons. Schafer illustrated how an increased demand is placed upon the cervical musculature when the cervical curve is straightened or reversed [50]. It is important that the cervical spine be in a normal structural alignment. A loss of the cervical lordosis and concomitant forward head posture may elicit the pelvo-ocular reflex, which causes an anterior pelvic translation to balance the head's center of gravity [51]. Wu et al [52,53] point out that in postural control, preference is given to the position of the head, neck, and trunk. Therefore, correction of the cervical spine becomes imperative so that the rest of the spine can be rehabilitated in relation to a normal reference point in space.

Once the cervical and lumbar lordoses are corrected, coronal reduction of the scoliotic curvatures begins. Here this was accomplished by adding a shoulderweight to the right shoulder and a hipweight to the anterior right ilium and posterior left ilium. Wu and Essien [53] have previously reported the effects of adding external weight to the upper body via a shoulder weight. They identified predictable patterns in which the trunk would compensate for the amount and position of the weight. Wu and MacLeod [52] identified a shift in the center of mass toward the added weight when placed on the side of the pelvis. However, the trunk and head remained in the same position, while the pelvis and lower extremities shifted to counteract the weight while supporting the head and trunk [52]. In this protocol, we created an environment where external weight was added to the head, shoulder, and pelvic regions simultaneously. Knowing the predictable patterns of compensatory shifting to an altered center of gravity, we placed the headweight, shoulderweight, and hipweights in areas designed to reduce each patient's specific spinal distortion patterns.

Learning a new motor coordination skill can be divided into 3 phases: cognitive, associative, and autonomous [54]. In the cognitive phase, the patient performs the motor task repetitively to learn until the task requirements are understood. As the patient progresses through the associative and autonomous phases, the task becomes easier to perform, and may ultimately be performed in a variety of practical contexts with decreased repetitions [54]. While Lantz et al [2] have shown that chiropractic management, consisting of a combination of manipulative procedures, electric stimulation, and orthotic inserts did not significantly reduce a scoliosis, this treatment does not incorporate these physiotherapeutic procedures. Instead, this treatment requires the use of specific rehabilitative equipment that theoretically recruits the use of head, neck, trunk, and extremity postural reflexes to create specific adaptation to an altered center of gravity and field of gaze.

The study design used here does present specific limitations. Due to the lack of a control group, comparative data and conclusions cannot be made. Additionally, a retrospective design does not blind the practitioners to treatment. Although we attempted to select patient files at random from 3 separate spine clinics, nonrandomized sample populations such as ours do not necessarily reflect the potential outcomes in a general population. Therefore, future studies in this area should incorporate a control group and a randomized patient population. Follow-up studies should also focus on the potential long-term benefits of conservative scoliosis treatment, given the relative scarcity of biomedical literature available on long-term benefits from any scoliosis treatment.

## Conclusions

Within the design limitations of the present study, the combined use of manipulative and neuromuscular rehabilitation seemed to reduce scoliotic curvatures in 19 subjects by an average of 17°. This reduction took place within a 4 to 6-week period. Although this treatment was not tested over the long term, the magnitude of the present results warrants further studies into its effectiveness. This treatment should also be tested on specific types of scoliosis in follow-up trials. A long-term investigation of this protocol is desirable.

## Competing interests

This manuscript was submitted by Spinal Technologies, a BioMed Central institutional member. The rehabilitation equipment used in this study is patented by Burl R Pettibon, DC and Spinal Technologies. MWM is the Director of Research for the Pettibon Biomechanics Institute, and an active postgraduate instructor for Spinal Technologies. MWM does not receive monetary compensation for this position. Rather, he is granted funding from Spinal Technologies to obtain biomedical literature and statistician services. DW is a past postgraduate instructor for Spinal Technologies, founder and director of the CLEAR Institute, and CEO of the Flex Neck Company. GL is an active postgraduate instructor for Spinal Technologies. The authors receive lecture fees for each continuing education seminar conducted. All 3 authors maintain private chiropractic practices from where all of the patient files in this study were taken. None of the above companies donated, funded, or reimbursed any monies or equipment for this study. None of the authors have any ownership in Spinal Technologies or its subsidiary companies, and none will gain any financial interest as a result of this paper.

## Authors' contributions

Each author worked on one-third of the patient population. The first author was responsible for collecting the data and putting our findings into written format.

## Pre-publication history

The pre-publication history for this paper can be accessed here:



## Figures and Tables

**Table 1:** Cobb Angle Measurements after 4–6 Weeks (Degrees)

**Subject #**	**Sex/Age**	**Initial**	**Post**	**Reduction**
1	F/19	32	24	8
2	M/17	23	8	15
3	F/15	29	11	18
4	F/64	52	19	33
5	F/16	19	4	15
6	F/22	28	11	17
7	F/27	16	2	14
8	F/34	25	11	14
9	F/21	35	20	15
10	F/41	28	13	15
11	F/53	40	22	18
12	F/18	31	9	22
13	F/16	27	14	13
14	F/20	33	7	26
15	F/23	32	18	14
16	F/15	16	4	12
17	M/33	15	4	11
18	M/24	21	6	15
19	F/25	38	11	17
